# Optimizing Surface Chemistry of PbS Colloidal Quantum Dot for Highly Efficient and Stable Solar Cells via Chemical Binding

**DOI:** 10.1002/advs.202003138

**Published:** 2020-11-27

**Authors:** Long Hu, Qi Lei, Xinwei Guan, Robert Patterson, Jianyu Yuan, Chun‐Ho Lin, Jiyun Kim, Xun Geng, Adnan Younis, Xianxin Wu, Xinfeng Liu, Tao Wan, Dewei Chu, Tom Wu, Shujuan Huang

**Affiliations:** ^1^ School of Materials Science and Engineering University of New South Wales (UNSW) Sydney NSW 2052 Australia; ^2^ School of Engineering Macquarie University Sustainable Energy Research Centre Macquarie University Sydney NSW 2109 Australia; ^3^ School of Photovoltaics and Renewable Energy Engineering University of New South Wales Sydney 2019 Australia; ^4^ Institute of Functional Nano and Soft Materials (FUNSOM) Soochow University Suzhou Jiangsu 215123 China; ^5^ Division of Nanophotonics CAS Key Laboratory of Standardization and Measurement for Nanotechnology CAS Center for Excellence in Nanoscience National Center for Nanoscience and Technology Beijing 100190 P. R. China

**Keywords:** chemical binding, PbS colloidal quantum dots, solar cells, surface chemistry

## Abstract

The surface chemistry of colloidal quantum dots (CQD) play a crucial role in fabricating highly efficient and stable solar cells. However, as‐synthesized PbS CQDs are significantly off‐stoichiometric and contain inhomogeneously distributed S and Pb atoms at the surface, which results in undercharged Pb atoms, dangling bonds of S atoms and uncapped sites, thus causing surface trap states. Moreover, conventional ligand exchange processes cannot efficiently eliminate these undesired atom configurations and defect sites. Here, potassium triiodide (KI_3_) additives are combined with conventional PbX_2_ matrix ligands to simultaneously eliminate the undercharged Pb species and dangling S sites via reacting with molecular I_2_ generated from the reversible reaction KI_3_ ⇌ I_2_ + KI. Meanwhile, high surface coverage shells on PbS CQDs are built via PbX_2_ and KI ligands. The implementation of KI_3_ additives remarkably suppresses the surface trap states and enhances the device stability due to the surface chemistry optimization. The resultant solar cells achieve the best power convention efficiency of 12.1% and retain 94% of its initial efficiency under 20 h continuous operation in air, while the control devices with KI additive deliver an efficiency of 11.0% and retains 87% of their initial efficiency under the same conditions.

## Graphical abstract

Ligand exchange is performed on PbS colloidal quantum dots using conventional PbX_2_ ligands and KI_3_ additives via a facile one‐step process, which simultaneously eliminate the undesirable sites and efficiently passivate the surface. The resulting solar cells achieve a power conversion efficiency of 12.1%.

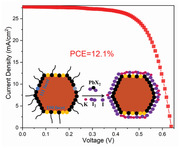

Colloidal quantum dots (CQDs) offer a promising platform to fabricate low‐cost, highly efficient, and stable photovoltaic devices due to their unique properties such as solution processability, size‐tunable bandgaps, and adjustable physical properties.^[^ ^1^, ^2^, ^3^, ^4^, ^5^, ^6^, ^7^, ^8^, ^9^, ^10^, ^11^, ^12^, ^13^ ^]^ Meanwhile, the multiple exciton generation effect in CQDs opens a possibility to break the Shockley–Queisser (S–Q) limit of single‐junction solar cells.^[^ ^14^, ^15^ ^]^ Benefiting from the device architecture design, band‐alignment engineering, and surface chemistry optimization, remarkable progress has been made in the past decade to improve the device performance and stability, while simplifying the fabrication process.^[^ ^16^, ^17^, ^18^, ^19^, ^20^, ^21^, ^22^ ^]^ Surface ligands of CQD are crucial to the surface chemistry since they determine the local charge states and transport properties, such as density of surface defect, carrier mobility, and energy band‐alignment.^[^ ^23^, ^24^, ^25^, ^26^, ^27^, ^28^, ^29^ ^]^ Driven by this unique feature of CQD, several strategies have been developed to modify CQD's surface chemistry via ligand exchange. Tang et al.^[^ ^30^ ^]^ and Chuang et al.^[^ ^31^ ^]^ developed atomic bromine and iodine to passivate PbS CQD surface, respectively. Subsequently, Liu et al.^[^ ^32^ ^]^ and Xu et al.^[^ ^33^ ^]^ improved the power conversion efficiency (PCE) of PbS CQD solar cell to over 11% and 12% by developing phase transfer ligand exchange and 2D matrix engineering for surface passivation of PbS CQD, respectively. Very recently, Sun et al. developed monolayer of perovskite bridging adjacent PbS CQD for fabricating solar cells, which achieved a record efficiency of 13.8%.^[^ ^34^ ^]^

To repair the surface defects before ligand exchange, post‐treatments were proposed to optimize the surface chemistry of as‐synthesized CQD. Ning et al.,^[^ ^35^ ^]^ Ip et al.,^[^ ^36^ ^]^ and Stavrinadis et al.^[^ ^37^ ^]^ restored undesirable surface sites of PbS CQD. Lan et al. utilized I_2_ molecules to remove unfavorable surface sites in 2016 and delivered a certified efficiency of 9.9%.^[^ ^38^ ^]^ Additionally, in our previous works, Zhang et al.^[^ ^39^ ^]^ and Hu et al.^[^ ^40^ ^]^ used perovskite quantum dots (QDs) to treat the PbSe CQDs via halide ion exchange mechanism, which achieved record efficiencies of 8.2% in 2017 and 9.2% in 2018, respectively. As demonstrated by the above results, suppression of surface defects in CQD led to significantly improved solar cell performance.

Crystallographic facet is another key factor governing the surface defects of CQDs. For PbS CQDs, (111) facets are terminated with Pb atoms and (100) facets are terminated with S and Pb atoms.^[^ ^41^, ^42^, ^43^ ^]^ Since as‐synthesized PbS CQDs are significantly off‐stoichiometric (lead‐rich), and S and Pb atoms distribute inhomogeneously,^[^ ^44^, ^45^, ^46^ ^]^ charge balance tends to be maintained via undercharged Pb species, which is a major cause of deep‐level traps.^[^ ^24^, ^47^ ^]^ In addition, inhomogeneous distribution of S and Pb atoms results in dangling bond of S atom in (100) facets, which is easily oxidized in air, leading to the formation of surface traps.^[^ ^24^, ^48^, ^49^, ^50^ ^]^ Further, the (100) facets might be insufficiently passivated during the ligand exchange process due to the small binding energy of ligand, thus resulting in nonuniform CQD aggregation.^[^ ^41^ ^]^ All these surface effects are detrimental to the CQD solar cell device performance. Meanwhile, surface chemistry also has a large influence on device stability due to the potential surface oxidation in the presence of molecular species such as oxygen and moisture, which was reported in numerous literature.^[^ ^51^, ^52^, ^53^ ^]^ Up to date, the realization of high efficiency CQD solar cell while maintaining its operational stability in air is still a big challenge. Although Cao et al.^[^ ^5^ ^]^ and Choi et al.^[^ ^54^ ^]^ significantly improved the light stability and operational stability of PbS CQD solar cells, respectively, new strategies are urgent to optimize the surface chemistry of CQDs to further enhance device performance and operational stability.

Here, we present a new strategy to simultaneously eliminate the undesirable sites and efficiently passivate the surface of PbS CQD via a facile one‐step process. In this process, the complex ligand comprising PbX_2_ (X = I and Br) matrix and potassium triiodide (KI_3_) additive was employed to optimize the surface chemistry. The aim is to combine the benefits from dissociative I_2_ and dissolved KI, both of which are generated from KI_3_ additive. The undercharged Pb species and dangling S sites were eliminated via reacting with dissociative I_2_ molecules and a robust high surface coverage shell on CQD was built by PbX_2_ and KI ligands to suppress the attack of moisture or oxygen. Consequently, the resultant PbS CQD solar cells with KI_3_ additive enable a higher efficiency of 12.1% and retain 94% of its initial efficiency under 20 h continuous operation in air while the control devices with KI additive delivers an efficiency of 11.4% and retain 87% of their initial efficiency under the same conditions.

PbS CQDs were synthesized and purified according to the reported procedure.^[^ ^8^, ^11^ ^]^ The purified PbS CQDs were ligand‐exchanged through the solution phase transfer method.^[^ ^32^ ^]^ Three types of solutions were prepared for ligand exchange. PbX_2_ control solution was prepared by dissolving PbI_2_, PbBr_2_, and NH_4_Ac into dimethylformamide (DMF) with concentrations of 0.1, 0.02, and 0.04 m, respectively. PbX_2_‐KI solution was prepared by adding KI additive into as‐prepared PbX_2_ control solution with a KI concentration of 0.01 m and PbX_2_‐KI_3_ solution was prepared by adding KI_3_ additive into as‐prepared PbX_2_ control solution with a KI_3_ concentration of 0.01 m. KI_3_ additive was prepared by dissolving the KI and I_2_ into DMF. Details can be found in Supporting Information. The reversible reaction KI + I_2_ ⇌ KI_3_ is fairly common in organic solvents, hence there are dissociative I_2_ molecules, KI, KI_3_, and PbX_2_ in the PbX_2_‐KI_3_ solution.^[^ ^55^ ^]^ The schematic diagram (**Figure** **1**a) depicts PbS CQD ligand exchange processes in three types of solutions, as shown in Process 1, 2, and 3. As‐synthesized PbS CQDs have two dominant facets, i.e., polar (111) facets with Pb atom termination and nonpolar (100) facets with S and Pb atom termination.^[^ ^38^, ^42^ ^]^ The undercharged Pb species are marked by Pb^0^ with blue spheres, as shown in Figure 1.

**Figure 1:**
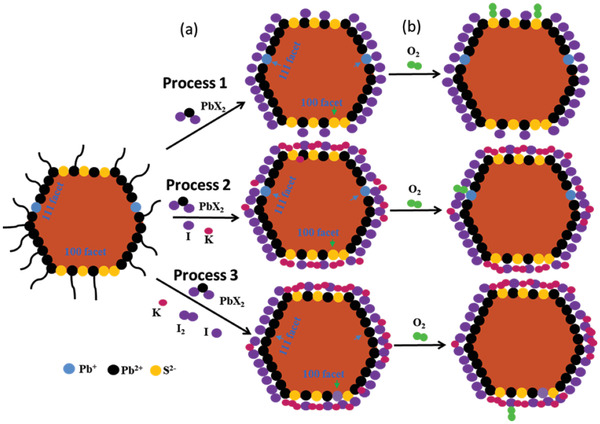
The hypothetical effects of a) ligand exchange processes in the three types of solutions and b) oxygen attack on ligand exchanged PbS CQDs. (The compared atoms are marked by the arrows during the ligand exchange procedure.)

During the ligand exchange process in the PbX_2_ control solution, the original oleic acid (OA) ligand would be replaced by PbX_2_. The Pb atoms would be bonded by iodine atoms of PbX_2_ due to electrostatic effect, which forms main shells on CQDs. However, the undercharged Pb species and dangling S atoms could not be eliminated as shown in Process 1.^[^ ^42^, ^53^, ^56^ ^]^ Meanwhile, the (100) facets could not be sufficiently capped, which was easily attacked by the oxygen and moisture, thus reducing their device performance and stability of solar cells.^[^ ^54^ ^]^ In the PbX_2_‐KI solution (Process 2), the undesirable Pb and S atoms remained, but uncapped surfaces would be passivated by the K atom. In the PbX_2_‐KI_3_ solution (Process 3), the undercharged Pb species and dangling S atoms would be eliminated by molecular I_2_ via the reaction of 2Pb^+^ + I_2_ = 2PbI^+^ and the reaction of PbS + I_2_ = PbI_2_ + S with large negative Gibbs free energy of −173.58 and −73.18 KJ mol^−1^,^[^ ^38^, ^53^, ^54^, ^57^ ^]^ respectively, and uncapped surfaces were also protected by K atoms.^[^ ^54^ ^]^ Therefore, the PbX_2_‐KI_3_ CQDs are expected to have the lowest defects and the highest stability among the three types of CQDs. Figure 1b schematically describes the attack of oxygen on ligand‐exchanged PbS CQDs. Oxygen could be adsorbed on the uncapped S sites and then oxidize S atoms in control PbS CQDs, thereby leading to surface defects, while the possible attacked sites could be effectively removed in the PbX_2_‐KI_3_ CQDs.

To verify our hypothesis, thorough characterizations were performed on the PbS‐PbX_2_‐KI_3_ CQDs, PbS‐PbX_2_‐KI CQDs, and PbS‐PbX_2_ control CQDs (as comparison with pristine OA‐capped PbS CQDs). Fourier transform infrared (FTIR) results show that the signal intensity of C—H modes at 2851 and 2921 cm^−1^ was dramatically reduced (Figure S1, Supporting Information), indicating that the insulating OA ligands of pristine PbS CQDs were successfully replaced with the corresponding ligands in all three types of films after ligand exchange.^[^ ^12^, ^58^ ^]^ Transmission electron microscopy (TEM) images show that the morphology and size of PbS‐PbX_2_‐KI_3_ CQDs have no significant change compared with PbS‐PbX_2_‐KI and pristine PbS‐OA CQDs (Figure S2, Supporting Information). X‐ray diffraction measurements confirmed that ligand exchanged PbS CQDs retain a rock salt without impurity phase, as shown in Figure S3 in the Supporting Information, indicating that KI_3_ addictive does not damage the PbS CQDs. This is also confirmed by a high‐resolution TEM image shown in Figure S2d in the Supporting Information.

The optical absorption spectra in **Figure** **2**a show that the exciton peak in the PbS‐PbX_2_‐KI_3_ CQD solution has a small redshift of 10 nm and slight peak broadening compared with the pristine PbS‐OA CQDs, while a larger redshift of 20 nm and peak broadening was observed in the PbS‐PbX_2_‐KI and PbS‐PbX_2_ CQD, suggesting that the PbS‐PbX_2_‐KI_3_ CQDs have fewer sub‐band detects than PbS‐PbX_2_‐KI and PbS‐PbX_2_ CQDs.^[^ ^59^, ^60^ ^]^ Steady‐state photoluminescence (PL) measurements were performed on PbS CQD, as shown in Figure 2b. The PL peak of PbS‐PbX_2_‐KI_3_ CQDs shows a small redshift of 10 nm and slightly broadening, whereas the PL peaks of PbS‐PbX_2_‐KI CQDs presents a larger shift of 20–25 nm and larger peak broadening compared with that of PbS‐OA CQDs. The half‐peak‐width in the PL peaks was calculated as ≈87, 89, 95, and 99 nm for the pristine PbS‐OA, PbS‐PbX_2_‐KI_3_, PbS‐PbX_2_‐KI, and PbS‐PbX_2_ CQDs, respectively, in consistence with the optical absorption results. Additionally, the PbS‐PbX_2_‐KI_3_ CQDs in solutions have the highest PL intensity, and the highest photoluminescence quantum yield of 15% (see Figure S4, Supporting Information), indicating that they have the least nonradiative recombination centers. Finally, the time‐resolved PL (TRPL) measurements were performed on the three ligand‐exchanged PbS QD films, as shown in Figure 2c, to investigate the dynamics of charge carrier recombination in the CQD solid films. The carrier lifetime was calculated to be 1.5 ns for the PbS‐PbX_2_‐KI_3_ film, 1.2 ns for the PbS‐PbX_2_‐KI film, and 0.9 ns for the PbS‐PbX_2_ control film by using the single‐exponential fitting to the TRPL curves. Overall, the optical characterizations demonstrate that PbS‐PbX_2_‐KI_3_ ligands improve CQD surface passivation as evidenced by the longest carrier lifetime and the lowest sub‐band defects, which is expected to facilitate the fabrication of highly efficient and stable solar cell.^[^ ^32^, ^60^ ^]^

**Figure 2:**
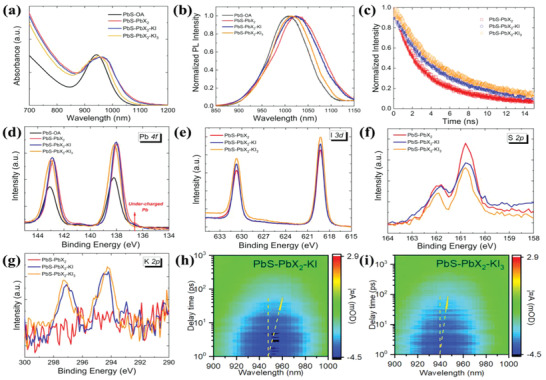
Characterizations of PbS CQDs. a) Optical absorption spectra and b) steady‐state PL characters of PbS CQD solution in mixed solvent of DMF and butylamine. c) TRPL curves for PbS CQD films. Core level XPS spectra of PbS CQD films in d) for I 3d, e) for Pb 4f, f) for S 2p, and f) for K 2p region. Spectrotemporal TAS maps of h) PbS‐PbX_2_‐KI control film and i) PbS‐PbX_2_‐KI_3_ target film.

CQD surface chemistry has a significant influence on their electronic properties, especially in terms of the carrier separation, transport, and recombination, which are key factors in photovoltaic devices.^[^ ^15^, ^24^ ^]^ X‐ray photoelectron spectroscopy (XPS) is a powerful tool to investigate the surface chemistry. First, it was found that the PbS‐PbX_2_‐KI_3_ film has the strongest peak intensity of I 3d (see Figure 2d), indicating that more iodine atoms are incorporated in the film. Second, as shown in Figure 2e, small peaks in the pristine PbS‐OA, PbS‐PbX_2_, and PbS‐PbX_2_‐KI films were observed at lower binding energy 136.8 eV, suggesting that there are undercharged Pb atoms, Pb^+^. Low binding energy peaks were not observed in the PbS‐PbX_2_‐KI_3_ film, indicating that undercharged Pb^+^ atoms in the PbS‐PbX_2_‐KI_3_ were removed by the reaction of 2Pb^+^ + I_2_ = 2PbI^+^ due to the addition of KI_3_ additives. In addition, the Pb 4f peaks of PbS‐PbX_2_‐KI_3_ film shift toward larger binding energy, indicating that undercoordinated Pb atoms on the surface reacted with I_2_ and formed PbI_2_, which is in line with previously reported results,^[^ ^24^, ^52^ ^]^ i.e., the binding energy of Pb—I bond is higher than that of Pb—S bond. Meanwhile, the narrowest peaks of S 2p in the PbS‐PbX_2_‐KI_3_ film (Figure 1f) indicate the most homogeneous local environment, which may arise from the elimination of dangling S atoms due to the formation of PbI_2_ shell via reaction PbS + I_2_ = PbI_2_ + S.^[^ ^52^, ^61^, ^62^ ^]^ Specifically, an atomic layer of PbI_2_ on PbS CQD surfaces could be formed by chemical binding, which suppresses the surface trap states and oxidation upon air exposure. In addition, the signals of K 2p were successfully probed in the PbS‐PbX_2_‐KI_3_ and PbS‐PbX_2_‐KI films while they were not acquired in the PbS‐PbX_2_ control film, as shown in Figure 2g, indicating the incorporation of K to PbS‐PbX_2_‐KI_3_ and PbS‐PbX_2_‐KI CQDs. The K incorporation on the S atoms could build robust shells on PbX_2_ uncapped sites, which blocks the attack of oxygen and moisture as well as improves the carrier mobility.^[^ ^54^ ^]^ The atomic ratio of three types of CQD films is given in Table S1 in the Supporting Information, which shows that the PbS‐PbX_2_‐KI_3_ CQDs have higher I/P ratio and lower O/Pb ratio (normalized to the Pb element), indicating that their surfaces were better passivated. The atomic force microscopy images as shown in Figure S5 in the Supporting Information reveal highly uniform surface morphology of three types solid films with an average roughness of about 1.0 nm. The pin–hole free and smooth morphology of films is expected to benefit the solar cell performance due to the suppression of current leakage at the junction interfaces.^[^ ^6^ ^]^ The optical images of three films are given in Figure S6 in the Supporting Information. Further, femtosecond transient absorption spectrum (fs‐TAS) measurements were carried out to investigate the charge transfer dynamics in the target and control films. Figure 2h,i shows the pseudo‐color TAS plots of both films excited at 600 nm, respectively. A ≈7 meV shift was observed for the PbS‐PbX_2_‐KI_3_ film whereas the shift is ≈11 meV for the control film. A small redshift of the transient bleach peak indicates less energy funneling. The reduced energy funneling in the target film evidences a flatter energy landscape and reduced tail states below the bandgap, thus benefiting photovoltaic cell with minimized *V*_oc_ deficit. Finally, To determine the trap‐state density, space charge limited current was measured by fabricating electron‐only devices (indium tin oxide (ITO)/PbS CQDs/Ag).^[^ ^63^, ^64^, ^65^ ^]^ The electron trap‐state densities (*N*_trap_) were calculated from the logarithm of the *J*–*V* curve measured in the dark by using the equation of *N*_trap_ = *V*_TFL_ (2*εε*_0_)/*ed* ^2^, where *d* is the film thickness and *e* and *ε*_0_ represent the elementary charge and vacuum permittivity respectively. The trap‐filled limit voltage (*V*_TFL_) is 0.746 V for the PbS‐PbX_2_‐KI_3_ film and 1.157 V for the PbS‐PbX_2_‐KI film. Therefore, the trap density of PbS‐PbX_2_‐KI_3_ film and PbS‐PbX_2_‐KI film is 1.03 × 10^16^ and 1.60 × 10^16^ cm^−3^, respectively. All above characterizations exhibit that the PbS‐PbX_2_‐KI_3_ CQDs have better surface passivation, which benefits for the resulting solar cell.

As evidenced in the above characterizations, the PbS‐PbX_2_‐KI_3_ approach provides the best surface passivation via formation of PbI_2_ shell on (111) facets and K atom shell on (100) facets. To further validate these passivation effects, we have fabricated PbS QD solar cells using these ligand exchange approaches. **Figure** **3**a shows the schematic diagram of device architecture where sol–gel processed Zn_0.9_Mg_0.1_O film serves as an electron transport layer according to the reference,^[^ ^6^ ^]^ one‐step deposited PbS CQD film acts as a light absorber layer and 1,2‐ethanedithiols (EDT) treated PbS CQD film serves as a hole transport layer. The cross‐sectional scanning electron microscope (SEM) image of the whole target device is given in Figure S8 in the Supporting Information, showing a Zn_0.9_Mg_0.1_O layer thickness of 70 nm, a PbS‐PbX_2_‐KI_3_ layer thickness of 400 nm and a PbS‐EDT layer thickness of 60 nm. The device performance was measured under AM1.5G 100 mW cm^−2^ illumination and the active area of solar cell is 0.070 cm^2^. Figure 3b shows the photocurrent *J*–*V* curves of the champion devices fabricated from three types of ligand exchanged films serving as light absorber layers. Apparently, the PbS‐PbX_2_‐KI_3_ device significantly outperforms both the PbS‐PbX_2_‐KI and PbS‐PbX_2_ devices and enables the highest efficiency of 12.1% together with an open‐circuit voltage (*V*_OC_) of 0.64, a short‐circuit current density (*J*_SC_) of 27.7 mA cm^−2^ and a fill factor (FF) of 0.68. The PbS‐PbX_2_‐KI device achieves an efficiency of 11.0% with a *V*_OC_ of 0.61 V, a *J*_SC_ of 27.2 mA cm^−2^, and an FF of 0.66. In contrast, the control PbS‐PbX_2_ device delivers the lowest efficiency of 10.4% with a *V*_OC_ of 0.60 V, a *J*_SC_ of 27.0 mA cm^−2^, and an FF of 0.64, which is comparable with the performance of reported devices.^[^ ^60^ ^]^ To confirm the *J*_SC_, external quantum efficiency (EQE) measurements were carried out on the three devices. As shown in Figure 2c, EQE is enhanced across the entire response region for the PbS‐PbX_2_‐KI_3_ device, resulting in an integrated *J*_SC_ of 27.1 mA cm^−2^, consistent with that from the *J–V* measurement. In addition, we have investigated the effect of the thickness of CQD absorbing layer on the device performance as shown in Table S2 in the Supporting Information, which shows that the optimal thickness of CQD absorbing layer is 360 nm for the PbS‐PbX_2_‐KI and PbS‐PbX_2_ solar cells, but 400 nm for the PbS‐PbX_2_‐KI_3_ solar cells, suggesting that photogenerated carriers in PbS‐PbX_2_‐KI_3_ solar cells could be collected more efficiently. The reproductivity of device performance is a crucial parameter and the PCE distribution of three types of devices is given in Figure 2d. The histogram of PCE for the 30 devices of each type confirms good reproducibility. The detailed parameters of device performance are given in **Table** **1**. Overall, PbS‐PbX_2_‐KI_3_ solar cells demonstrate the highest average efficiency of 11.8% with higher *V*_OC_ and FF, confirming that KI_3_ additive in ligand exchange process produces the best surface chemistry among the three ligand exchange approaches.

**Figure 3:**
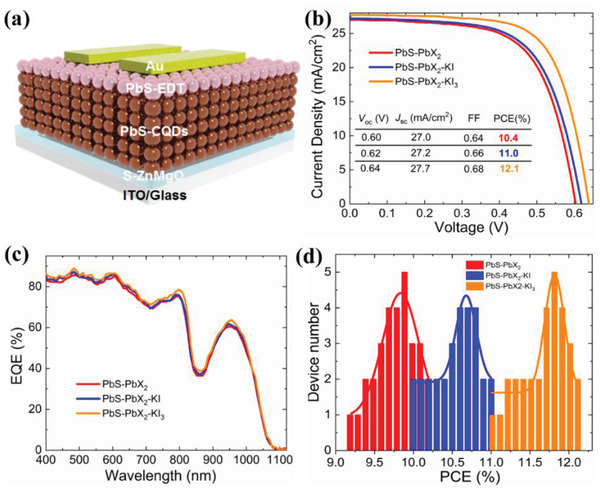
a) Schematic diagram of device architecture, b) *J–V* curves of the champion the three types of devices (inset: detailed values of device efficiency). c) EQE curves of champion PbS‐PbX_2_‐KI_3_, PbS‐PbX_2_‐KI, and PbS‐PbX_2_ devices. The integrated *J*_SC_ is 27.1 mA cm^−2^ for the PbS‐PbX_2_‐KI_3_ device, 26.5 mA cm^−2^ for the PbS‐PbX_2_‐KI device, and 26.1 mA cm^−2^ for the PbS‐PbX_2_ device. d) PCE distribution of PbS CQD devices based on 30 samples for each approach.

**Table 1:** Statistics for device performance for PbS CQD solar cells (30 samples for each approach)

Device type	*V*_OC_ [V]	*J*_SC_ [mA cm^−2^]	FF	PCE [%]
PbS‐PbX_2_ device	0.58 ± 0.04	26.5 ± 3.6	0.62 ± 0.06	9.6 ± 0.4
PbS‐PbX_2_‐KI device	0.60 ± 0.04	26.8 ± 1.2	0.66 ± 0.05	10.6 ± 0.3
PbS‐PbX_2_‐KI_3_ device	0.63 ± 0.03	27.5 ± 1.0	0.68 ± 0.05	11.8 ± 0.3

To further understand the impact of surface passivation on device operation, device physical characterizations were performed on the PbS‐PbX_2_‐KI_3_ and PbS‐PbX_2_‐KI devices. First, capacitance–voltage (*C*–*V*) test was used to determine built‐in potential (*V*_bi_) and the depletion width in the solar cells. **Figure** **4**a shows the Mott–Schottky curves of the two solar cells, which show that the *V*_bi_ of the PbS‐PbX_2_‐KI_3_ device is 0.68 V, whereas *V*_bi_ of PbS‐PbX_2_‐KI device is 0.64 V. The larger *V*_bi_ in the PbS‐PbX_2_‐KI_3_ device indicates a stronger built‐in field, beneficial for charge carrier collection. Figure 4b shows the calculated depletion width of the PbS‐PbX_2_‐KI_3_ device and PbS‐PbX_2_‐KI device as a function of applied voltage. The PbS‐PbX_2_‐KI_3_ device has a larger width of 300 nm, while the PbS‐PbX_2_‐KI device has a width of 268 nm under the short‐circuit condition. The larger depletion width results from improved *V*_bi_ and reduced surface trap density. In addition, the dependence of *V*_OC_ and *J*_SC_ on light intensity was investigated to elucidate the charge carrier generation and recombination mechanisms. As shown in Figure 4c, the data of *J*_SC_ versus light intensity was fitted using *J*_SC_ ∝ *I^*α*^*, where *I* is the light intensity and *α* is an exponent factor. The calculated *α* for the PbS‐PbX_2_‐KI_3_ and PbS‐PbX_2_‐KI solar cells is 0.98 and 0.96, respectively. The slightly higher value of the target device indicates weaker charge recombination at higher light intensity.^[^ ^11^, ^40^ ^]^ In Figure 4d, the light intensity dependence on *V*_OC_ was also studied following the relation: *V*_OC_ ∝ *ηkT/q*, which depends on the charge recombination mechanism in solar cell. Here *K*, *T*, and *q* are the Boltzmann constant, temperature in Kelvin, and the elemental charge, respectively. In a CQD heterojunction solar cell, larger *η* value indicates the presence of more defect trap states, which will affect the rate of trap‐assisted charge recombination. The calculated slopes are 1.05 *KT/q* for the PbS‐PbX_2_‐KI_3_ solar cell, smaller than that of 1.10 *KT/q* for the PbS‐PbX_2_‐KI solar cell, suggesting that the target solar cell possesses reduced sub‐bandgap trap recombination.^[^ ^11^, ^40^ ^]^ Finally, Figure 4e,f shows the normalized transient photocurrent (TPC) and transient photovoltage (TPV) decay of the PbS‐PbI_2_‐KI_3_ and PbS‐PbX_2_‐KI solar cell, respectively. Here we use a single exponential function to fit the TPV decay curves and extract the electron lifetime. TPC results show that the PbS‐PbX_2_‐KI_3_ solar cell has a shorter lifetime of 41 µs, while the PbS‐PbX_2_‐KI solar cell has a lifetime of 46 µs, indicating that the PbS‐PbX_2_‐KI_3_ solar cell has a higher carrier transport rate. TPV results exhibit that the PbS‐PbX_2_‐KI_3_ solar cell has a longer lifetime of 34 µs, while PbS‐PbX_2_‐KI solar cell has a lifetime of 27 µs, indicating that PbS‐PbX_2_‐KI_3_ solar cell has a slower carrier recombination rate. TPC and TPV results demonstrate that the carrier collection efficiency in PbS‐PbX_2_‐KI_3_ solar cell was improved compared with that in PbS‐PbX_2_‐KI solar cell.

**Figure 4:**
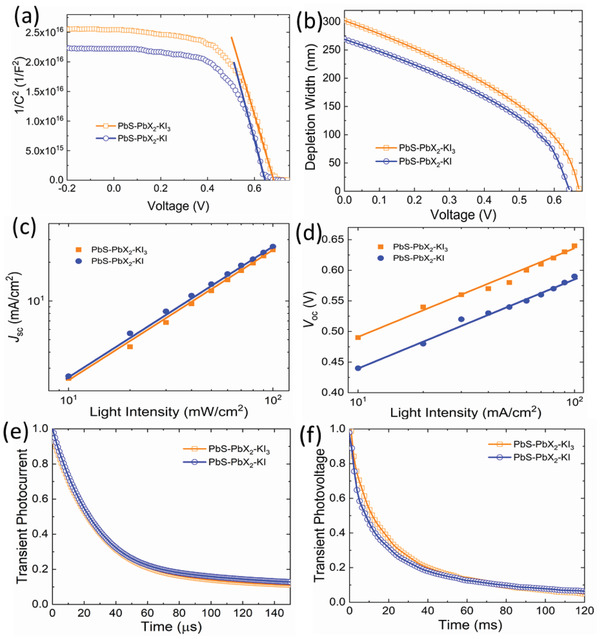
Characterization of the target and control PbS CQD solar cells. a) *C*–*V* curves from which built‐in potential *V*_bi_ is estimated by the intercept to the *x*‐axis. b) Depletion width as a function of applied voltage. c) *V*_OC_ and d) *J*_SC_ of PbS‐PbX_2_‐KI_3_ and PbS‐PbX_2_‐KI solar cells as a function of incident light intensity. e) TPC decay spectroscopy and f) TPV decay spectroscopy of PbS‐PbX_2_‐KI_3_ and PbS‐PbX_2_‐KI solar cells.

To evaluate the effect of surface chemistry on film stability, we first studied the thermal stability of the PbS‐PbX_2_‐KI_3_ and PbS‐PbX_2_‐KI films under 85 °C heat treatment for 0–20 h in air via light absorption measurements. As shown in **Figure** **5**a,b, no obvious shifts of the exciton peak in the films were observed, suggesting that there was negligible oxidation under the continuous heating.^[^ ^54^ ^]^ This evidenced that the CQD's surface was robustly passivated by the PbX_2_ and K shells, which resisted the attack of oxygen or moisture. However, half‐peak‐width of the exciton peaks become broader, indicating that some PbS CQDs in both films may merge together, resulting in nonuniform size distribution hence peak broadening.^[^ ^41^ ^]^ However, the peak in the PbS‐PbX_2_‐KI_3_ film was significantly narrower than that of the control film, indicating that the PbS‐PbX_2_‐KI_3_ CQDs own stronger resistance to high temperature because of the removal of undercoordinated Pb and dangling bond S atoms. Second, XPS measurements were performed on both films before and after continuous annealing to examine oxygen content. Figure 5c shows oxygen to lead atomic ratio of both films before and after annealing for 20 h in air. It is found that the PbS‐PbX_2_‐KI_3_ films retain more iodine and less oxides on their surfaces than PbS‐PbX_2_‐KI films^[^ ^54^ ^]^ (green histogram: before heat treatment; purple histogram: after heat treatment). These indicate that PbS‐PbX_2_‐KI_3_ CQDs have stronger antioxidation ability. Finally, the device operation stability was tested by measuring *J–V* curve after 20 h operation at maximum power point (MPP) in ambient conditions, as shown in Figure 5d. We observed less performance loss of the PbS‐PbX_2_‐KI_3_ device, retaining 94% of its initial efficiency, whereas the control devices retained 87% of their initial performance. Before the operation test, the PbS‐PbX_2_‐KI_3_ device demonstrated PCE of 11.9% together with a *V*_OC_ of 0.64 V, a *J*_SC_ of 27.3 mA cm^−2^, and an FF of .68, and the PbS‐PbX_2_‐KI device delivered a PCE of 11.0% together with a *V*_OC_ of 0.62 V, a *J*_SC_ of 26.9 mA cm^−2^, and an FF of 0.66. After the operation test, the former device retained a PCE of 11.2%, only net 0.7% PCE decrease while the latter device retained a PCE of 9.6%, net 1.4% PCE decrease (see inset table of Figure 5d). These results demonstrated that PbS‐PbX_2_‐KI_3_ films enhance their device operation stability due to better surface chemistry.

**Figure 5:**
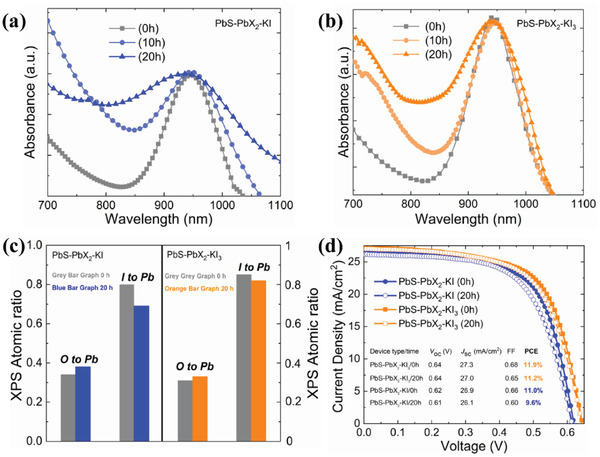
Optical absorption spectra of a) PbS‐PbX_2_‐KI film b) PbS‐PbX_2_‐KI_3_ film under continuous heat treatment in air. c) Atomic ratios of the films before and after continuous annealing. d) *J–V* curves of the devices before and after continuous operation under 1‐sun conditions at MPP in air.

In summary, we have developed a new strategy to optimize the surface passivation of PbS CQD via eliminating the undercharged Pb atoms and dangling bond of S sites, simultaneously building robust PbX_2_ and K shells on PbS CQDs during the ligand exchange process by KI_3_ additives. As a result, the surface trap states of the CQDs have been significantly reduced and the thermal stability has been enhanced. The solar cells made with KI_3_ addition achieved an efficiency of 12.1% with enhanced operational stability. In addition, this work comprehensively demonstrates the important effect of additives for optimizing the CQD surface chemistry, which provides a crucial guideline for applications of other quantum dot and nanoparticle materials.

## Experimental Section

##### Materials

Lead oxide (PbO), OA, 1‐octadecene (ODE), hexamethyldisilathiane (TMS), lead iodine (PbI_2_), lead bromide (PbBr_2_), potassium iodide (KI), iodine molecule (I_2_), ammonium acetate (NH_4_Ac), *N*,*N*‐DMF, 2‐methoxyethanol, monoethanolamine (MEA), zinc acetate dihydrate, and magnesium acetate tetrahydrate were purchased from the Sigma.

##### Synthesis of PbS CQDs

0.9 g lead oxide (PbO), 2.9 g oleic acid, and 20 mL ODE were loaded into a three‐neck flask and pumped under continuous stirring at 95 °C for 6 h to form transparent and colorless Pb precursor solution. Next, 280 µL TMS in 10 mL degassed ODE was quickly injected into Pb precursor at 90 °C and heated for 10 s. Then the heating was turned off (heating mantle removed) and the solution was cooled to room temperature naturally. The PbS CQDs were purified three times by precipitation using acetone and centrifugation at 5000 rpm for 30 s according with the reference.^[^ ^6^, ^8^ ^]^

##### Ligand Exchange

KI was added into 100 mL DMF to form mother solution with a concentration of 0.01 m (KI‐mother solution) and molecular I_2_ was added into 100 mL DMF to prepare mother solvent with a concentration of 0.001 m (I_2_‐mother solution). Then, 2 mL I_2_‐mother was added into 20 mL KI‐mother solution to obtain KI_3_ mother solution. Lead iodide (0.1 m PbI_2_), lead bromide (0.02 m PbBr_2_), and NH_4_Ac (0.04 m) were dissolved in DMF solution to prepare PbX_2_ solution. KI mother solution and KI_3_ mother solution were added into PbX_2_ solution to prepare PbX_2_‐KI and PbX_2_‐KI_3_ solutions, respectively. 5 mL of above purified PbS CQDs in octane (10 mg mL^−1^) was introduced into the three types as‐prepared ligand exchange solution. Following 2 min vortexing of the solution, CQDs in octane transferred into DMF, indicating ligand exchange process completed. The ligand exchanged CQD solutions were then washed three times using octane to remove organic residues. The ligand exchanged CQDs were collected by precipitating with the addition of toluene and drying under vacuum for 20 min. The obtained ligand exchanged PbS CQDs were dispersed into mixed solvent of DMF and butylamine with a volume ratio of 3:7 to form CQD ink with a concentration of 500 mg mL^−1^. All the ligand exchange procedures were performed under ambient conditions.

##### Preparation of Zn_0.9_Mg_0.1_O Precursor Solution

The precursor was prepared by using zinc acetate dihydrate and magnesium acetate tetrahydrate as the Zn and Mg source, respectively. 2‐Methoxyethanol and MEA were employed as solvent complexant, respectively. The molar ratio of MEA to metal ions (0.35 m) is 1:1. The solution was continuously stirred at 60 °C for 24 h to produce a clear and homogenous sol with a yellowish color.

##### Device Fabrication

All devices were fabricated under ambient conditions. ITO glass was cleaned by detergent, deionized water water, and acetone and then treated with oxygen plasma. Zn_0.9_Mg_0.1_O precursor solution was spin coated on ITO glass and annealed at 300 °C in air for 30 min. Then corresponding PbS inks were spin coated on the Zn_0.9_Mg_0.1_O films to deposit the light absorber layers. Subsequently, hole transport layer, i.e., EDT treated PbS QD layer was deposited by conventional layer‐by‐layer deposition.^[^ ^54^ ^]^ Finally, 100 nm gold electrodes were fabricated by thermal evaporation.

##### Characterizations

The PL spectra measurements were carried out at room temperature using a homemade laser PL spectroscopy system (Crystal Laser, Model BLC‐050‐405). The laser pulse width was 130 fs and the repetition rate was 100 m Hz. The excitation wavelength for both PL and TRPL measurements is 600 nm. For PL quantum yield measurements, an integrated sphere was used. SEM measurements were carried out by using an FEI Nova Nano SEM 450. TEM measurements were performed by a JEOL JEM‐F200 operated at 200 kV. UV–vis absorption spectra were recorded using U‐4100 spectrophotometer (Hitachi). FTIR spectroscopy was performed on a Thermo Fisher FTIR6700. XPS measurements were conducted by a VG ESCALAB MK2 system with monochromatized Al *K*_*α*_ radiation under a pressure of 5.0 × 10^−7^ Pa. The space‐charged limited current method was used to determine the surface trap density.

The current density–voltage (*J–V*) characteristics of devices were measured using Keithley 2400 (*I*–*V*) digital source meter under a simulated AM 1.5G solar irradiation at 100 mW cm^−2^ (Newport, AAA solar simulator, 94023A‐U). The EQE spectra were recorded through certified EQE equipment by using a photovoltaic Measurement QXE7 Spectral Response system with monochromatic light generated from a xenon arc lamp. The system was calibrated by a silicon diode. The operation stability test at continuous MPP operation under 1 sun, AM 1.5G illumination was studied in an air ambient (40% ± 10% relative humidity) at room temperature by fixing the voltage at voltage of maximum power point. The devices were biased with white light to reach near *V*_oc_ conditions and photoexcited with low power laser (500 nm) pulses to generate small photovoltage perturbations (∆*V* kept to lower than 30 mV) to obtain transient photovoltage and photocurrent results. Single‐exponential fits to normalized traces were used to estimate the carrier lifetime at *V*_oc_ conditions. Capacitance–voltage (*C*–*V*) measurements were carried out using an analyzer under dark conditions with an AC signal set at 1 kHz frequency and 50 mV amplitude.

## Conflict of Interest

The authors declare no conflict of interest.

## Supporting information

Supporting Information
