# Supplementary material for: Optimizing Surface Chemistry of PbS Colloidal Quantum Dot for Highly Efficient and Stable Solar Cells via Chemical Binding
Source: Adv Sci (Weinh). 2020 Nov 27;8(2):2003138. doi: 10.1002/advs.202003138 (PMC7816699; doi:10.1002/advs.202003138)
Supplement: Supplementary file 1 — Supporting Information [file ADVS-8-2003138-s001.pdf]

© 2020 Wiley-VCH GmbH

ADVANCED SCIENCE
Open Access

Supporting Information

for *Adv. Sci.*, DOI: 10.1002/advs.202003138

Optimizing Surface Chemistry of PbS Colloidal

Quantum Dot for Highly Efficient and

Stable Solar Cells via Chemical Binding

Long Hu, Xinwei Guan, Qi Lei, Robert Patterson, Jianyu Yuan, Chun-Ho Lin, Jiyun Kim, Xun Geng, Adnan Younis, Xianxin Wu, Xinfeng Liu, Tao Wan, Dewei Chu, Tom Wu, and Shujuan Huang**

SUPPLEMENTARY INFORMATION

Optimizing Surface Chemistry of PbS Colloidal Quantum Dot for Highly Efficient and Stable Solar Cells via Chemical Binding

Long Hu[a, b], Xinwei Guan[a], Qi Lei[a], Robert Patterson[c], Jianyu Yuan[d], Chun-Ho Lin[a], Jiyun Kim[a], Xun Geng[a], Adnan Younis[a], Tao Wan[a], Dewei Chu[a], Tom Wu[a]* and Shujuan Huang[b]*

Dr. L. Hu, Q. Lei, X. Guan, Dr. C. Lin, J. Kim, X. Geng, Dr. A. Younis, Dr. T, Wan, A/Prof. D. Chu, Prof. T. Wu.

[a] School of Materials Science and Engineering, University of New South Wales (UNSW), Sydney, NSW, 2052, Australia.

Email: tom.wu@unsw.edu.au

Dr. L. Hu, A/Prof. S. Huang.

[b] School of Engineering, Macquarie University Sustainable Energy Research Centre, Macquarie University, Sydney, NSW, 2109, Australia.

Email: Shujuan.huang@mq.edu.au

Dr. R. Patterson.

[c] School of Photovoltaics and Renewable Energy Engineering, University of New South Wales, Sydney, Australia.

A/Prof. J. Yuan.

[d] Institute of Functional Nano & Soft Materials (FUNSOM), Soochow University, Suzhou, Jiangsu 215123, China.

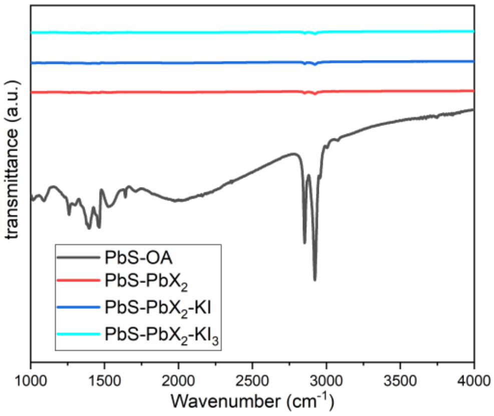

Figure S1. FT-IR spectra of CQD films before (PbS-OA) and after ligand exchange. No peaks of organic ligands were observed after ligand exchange, indicating OA ligand removal after the solution-phase ligand exchange in the three films.

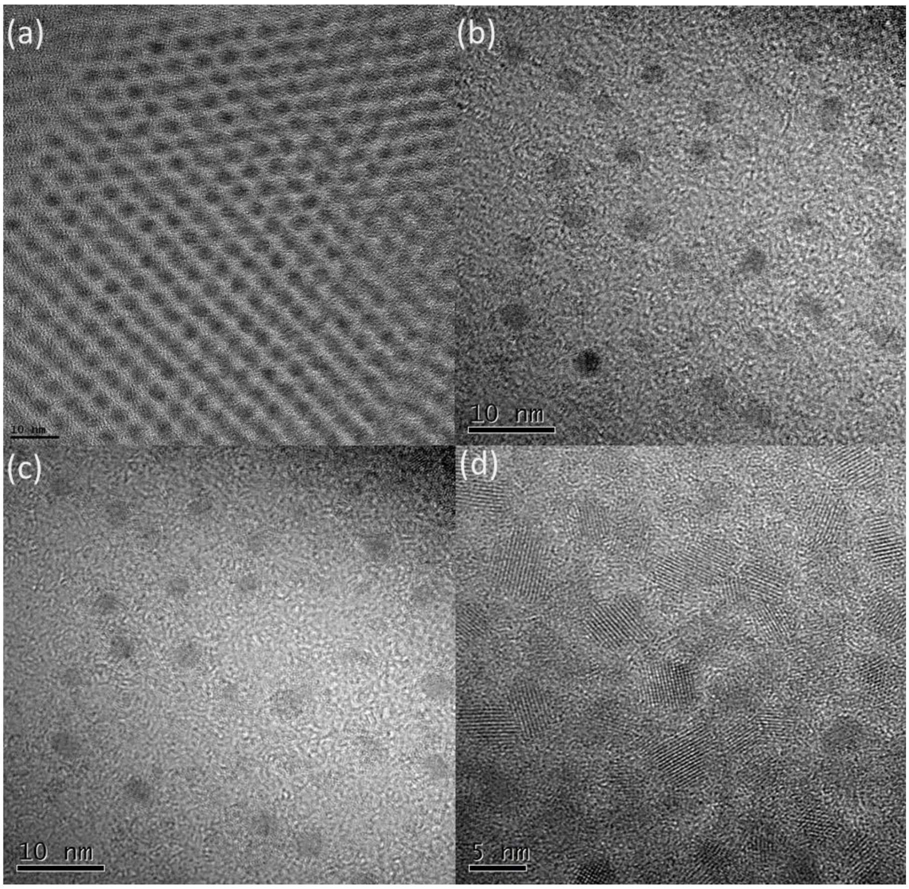

Figure S2. TEM images of (a) PbS-OA, (b) PbS-PbX_2-KI, and (c) PbS-PbX_2- KI_3 PbS CQDs. (d) High-resolution TEM image of PbS-PbX_2- KI_3 PbS CQDs. No changes of size, shape and crystalline structure were observed after ligand exchange.

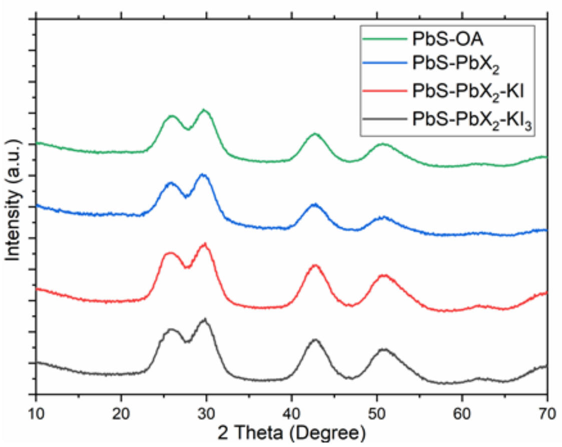

Figure S3. XRD patterns of PbS-PbX_2- KI_3, PbS-PbX_2- KI, PbS-PbX_2 and PbS-OA films.

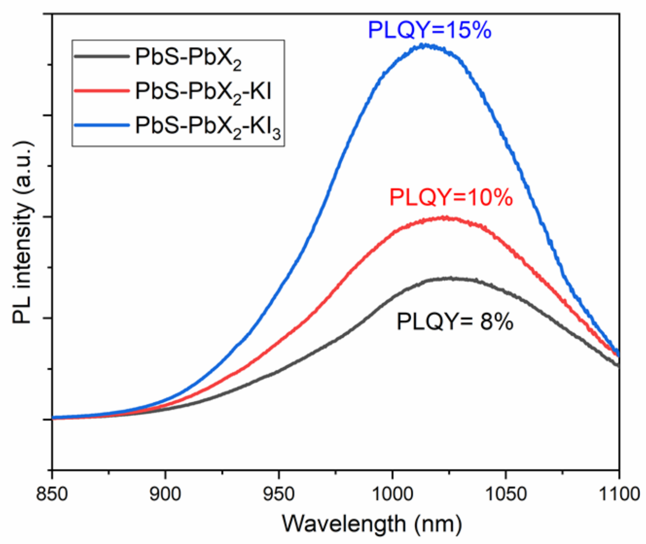

Figure S4. PL intensity and PLQY of PbS-PbX2-KI$_3$, PbS-PbX_2-KI and PbS-PbX_2 CQDs.

Table S1. The atomic ratio of three types of CQD films (Normalized to the Pb element).

Elemental ratio	PbS-PbX_2-KI_3	PbS-PbX_2-KI	PbS-PbX_2
I/Pb	60.7%	55.6%	55.1%
S/Pb	67.9%	71.1%	72.0%
O/Pb	20.4%	23.6%	24.1%

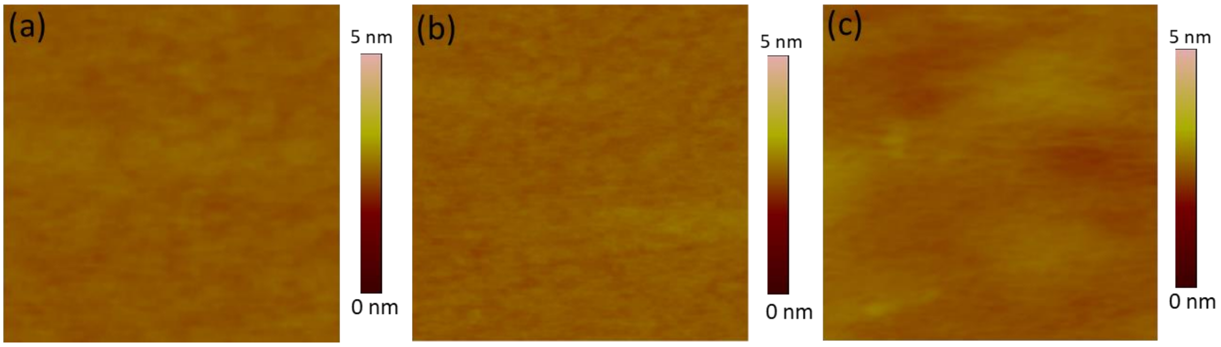

Figure S5. AFM image of (a) PbS-PbX_2-KI_3 film (b) PbS-PbX_2-KI film (c) PbS-PbX_2 film. (5 x 5 um)

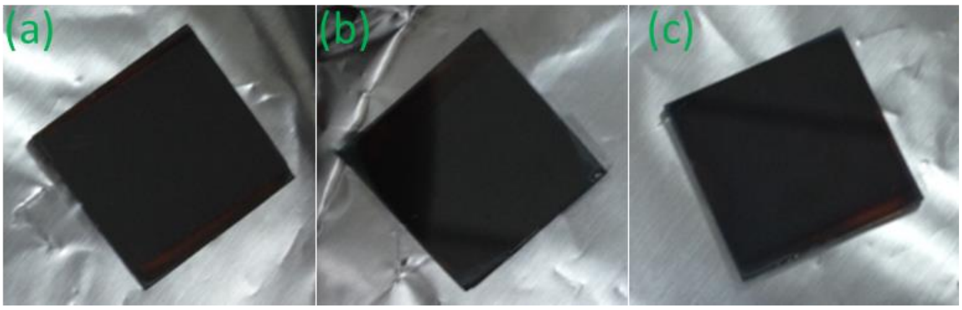

Figure S6. Optical images of (a) PbS-PbX_2-KI_3 (b) PbS-PbX_2-KI and (c) PbS-PbX_2 films.

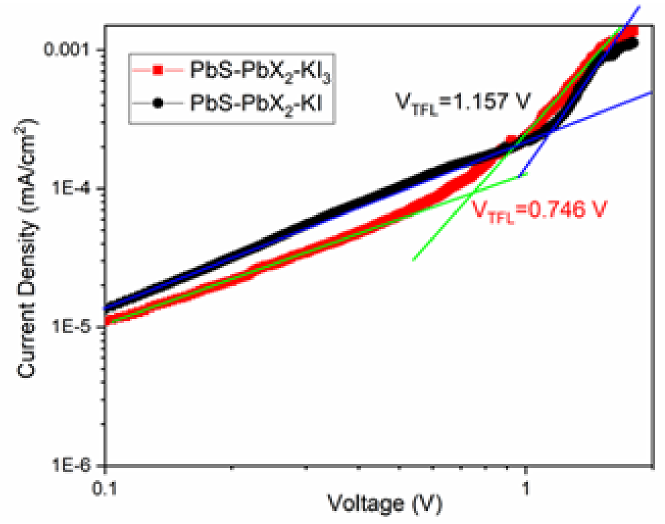

Figure S7. Logarithm of the *I–V* curve in the dark.

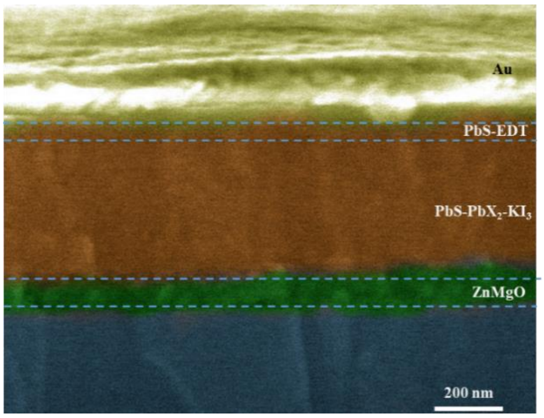

Figure S8. Cross-sectional SEM image of PbS-PbX_2-KI_3 device consisting of ITO/ZnMgO/PbS-PbX_2-KI_3/PbS-EDT/Au.

Table S2. The device performance in terms of the thickness of CQD absorbing layer.

Thickness	Average PCE (%)		
	$PbS-PbX_2$ device	$PbS-PbX_2$-KI device	$PbS-PbX_2-KI_3$ device
310 nm	9.1 ± 0.5	9.4 ± 0.5	9.5 ± 0.5
360 nm	9.8 ± 0.4%	10.6 ± 0.4	10.9 ± 0.5
400 nm	9.6 ± 0.4%	10.6 ± 0.3	11.6 ± 0.4
450 nm	9.0 ± 0.4%	10.1 ± 0.3	11.4 ± 0.5
